# An 8-Week Ketogenic Low Carbohydrate, High Fat Diet Enhanced Exhaustive Exercise Capacity in Mice

**DOI:** 10.3390/nu10060673

**Published:** 2018-05-25

**Authors:** Sihui Ma, Qingyi Huang, Koichi Yada, Chunhong Liu, Katsuhiko Suzuki

**Affiliations:** 1 Graduate School of Sport Sciences, Waseda University, Tokorozawa 359-1192, Japan; masihui@toki.waseda.jp (S.M.); hqyaaaaaa@163.com (Q.H.); 2 College of Food Science, South China Agricultural University, Guangzhou 510642, China; 3 The Key Laboratory of Food Quality and Safety of Guangdong Province, Guangzhou 510642, China; 4 Research Organization for Nano and Life Innovation, Waseda University, Tokorozawa 359-1192, Japan; yada_koich@yahoo.co.jp; 5 Faculty of Sport Sciences, Waseda University, Tokorozawa 359-1192, Japan

**Keywords:** ketogenic diet, keto-adaptation, endurance exercise capacity, muscle damage

## Abstract

Current fueling tactics for endurance exercise encourage athletes to ingest a high carbohydrate diet. However, athletes are not generally encouraged to use fat, the largest energy reserve in the human body. A low carbohydrate, high fat ketogenic diet (KD) is a nutritional approach ensuring that the body utilizes lipids. Although KD has been associated with weight-loss, enhanced fat utilization in muscle and other beneficial effects, there is currently no clear proof whether it could lead to performance advantage. To evaluate the effects of KD on endurance exercise capacity, we studied the performance of mice subjected to a running model after consuming KD for eight weeks. Weight dropped dramatically in KD-feeding mice, even though they ate more calories. KD-feeding mice showed enhanced running time without aggravated muscle injury. Blood biochemistry and correlation analysis indicated the potential mechanism is likely to be a keto-adaptation enhanced capacity to transport and metabolize fat. KD also showed a potential preventive effect on organ injury caused by acute exercise, although KD failed to exert protection from muscle injury. Ultimately, KD may contribute to prolonged exercise capacity.

## 1. Introduction

Fat has played a dominant role in human life since the antediluvian period, where primary fuel came from meat and fat gathered or hunted by hunters. However, agriculture shifted meat-consumption to mainly starch consumption. Human calorie intake thus changed from fat into carbohydrates, since large quantities of farm products could be obtained using cultivating techniques.

Modern food preserving techniques such as freezing, canning and irradiation extend the shelf life of food, but in the long term, the human body still stores energy in the form of fat.

A classic definition of ketogenic diet (KD) is a nutritional approach consisting of hyper-fat, an adequate amount of protein but insufficient level of carbohydrates, which means the share of glucose-induced metabolic activities should constitute less than 5% of daily calorie intake, or for humans, ~20 g per day [1,2]. KD is an effective implementation of fat-adaptation. The below-average amount of carbohydrates failed to maintain the circulation of oxaloacetate or satisfy the demand of Kreb’s cycle; therefore, neither glucose oxidation nor fat oxidation could continue its adequate supply. In this situation, the energy supply system shifted into ketone bodies (KB), including acetoacetate (AcAc), 3-hydroxybutyrate (3-HB), and a small amount of acetone, all of which are generated from ketogenesis, a process converse acetyl-CoA, synthesized in mitochondria by β-oxidation of fatty acids, the main energy source during ketogenic diet. KB could help mitochondria to produce more ATP, thus an adapted utilization of KB released more available energy than glucose utilization. This successful adaptation of KB utilization was firstly used by Krebs, he termed it as “physiological ketosis” [3].

Due to modern science, a considerable amount is now known about metabolism during starvation, satiety, rest and exercise [4]. Experiencing physiological ketosis means that the body becomes successful at fat utilization, indicating reduced lipogenesis and increased lipolysis and fat oxidation. Benefiting from above, effect of KD on weight control is undeniably effective [5,6,7,8,9,10].

Since fat reserves are suggested as a potentially rich source of energy for exercise, many attempts have been made to harness fat-utilization as a strategy to improve exercise performance. However, studies investigating KD in exercise capacity have shown contradictory results [11,12,13,14,15,16,17,18,19,20,21,22,23,24,25,26,27,28].

Decades ago, several ketogenic, fat utilization and adaptation trials failed to enhance exercise performance in human individuals. Back to 1970, Pruett and colleagues assessed how standard diets (31% fat), fat-enriched diets (64% fat) or carbohydrate-enriched diets (8% fat, 82% carbohydrate) contributed to endurance bout test. In this study, adaptation to fat failed to enhance exercise capacity [11]. In 1983, Phinney and colleagues conducted a 4-week fat-adaptation and cycling capacity test trial in human individuals [12]. Though they failed to increase the endurance capacity in these studies, they expanded horizons for study into low carbohydrate, high fat diets (LCHF) or KD application to athlete diets.

Researches focused on LCHF or KD for endurance capacity were carried out afterwards, but these researches achieved mixed conclusions. They were all undertaken on trained athletes. Two of them managed to enhance endurance capacity, but their experimental designs have obvious flaws [13,14]. In Muoio’s study, diet was not administered randomly, while the fat percentage was relatively low (38% of total energy intake). In both Muoio and Lamberts’ studies, more than one exercise test were performed, but the intervals between them were very short, which may have affected the results. However, in 1999, when Lambert et al. once again conducted a fat-adaptation study in endurance cyclists, they found a 5~10-day fat ingestion could contribute to substrate mobilization without significant impact on exercise performance in 40-km cycling [15]. The door of fat-loading has been shut once, as Burke et al., putting the conclusion as “a nail in the coffin” after all these trials lead a clear enhancement in athletes [16]. However, a subsequent paper by Burke et al. suggested that it was important to have another look at this topic [17]. Consequently, new claims currently discuss the feasibility of fat adaptation on enhancement of sports performance. The study of LCHF or KD application in athletes was on its way to reaching a second place [17,18,19,20,21,22].

It is clearly interesting to find a possible connection between fat adaptation and exercise performance. Consequently, some studies conducted in humans have provided interesting results.

Wycherley and colleagues showed that KD enhanced exercise capacity in untrained human subjects in an article published in 2007 [23]. In 2014, Zajac and colleagues reported that KD increased VO_2_ max and improved lactate threshold in off-road cyclists [24]. In 2016, researchers from United Kingdom and United States of America reported that cycling time was improved by nutritional ketosis [25]. In 2018, McSwiney et al. found that keto adaptation enhanced six-second sprint and critical power tests in well-trained athletes [26]. On the contrary, in a study in 2017, conducted by Zinn et al., in a pilot study of New Zealand endurance athletes, KD failed to enhance endurance capacity along with an increased benefit in body composition and well-being [27]. Meanwhile, LCHF showed impairment of exercise economy and performance benefit from intensified training in a group of elite race walkers, according to a research conducted in 2017 [28]. On the other hand, White and colleagues reported an increase of perception of fatigue and a direct relation between blood KB and fatigue by KD [29].

Human trials are always restricted due to various reasons, whereas animal experiments are capable for wider testing. “The proper study of mankind is man” is a famous declaration by Alexander Pope. To investigate the effect of KD on endurance exercise capacity in depth, we designed a two-month KD-adapting experiment in mice, after which we conducted a single-blind maximum endurance capacity test (MECT) using the treadmill and undertook blood biochemistry and tissue analyses.

## 2. Materials and Methods

### 2.1. Mouse Maintenance and Diets

Male C57BL/6J mice (*n* = 35) were purchased from Takasugi Experimental Animals Supply (Kasukabe, Japan) at 7 weeks of age. Four or five animals were housed together in 1 cage (27 × 17 × 13 cm) in a controlled environment under a light–dark cycle (lights on at 0800 and off at 2000). The experimental procedures followed the Guiding Principles for the Care and Use of Animals in the Academic Research Ethical Review Committee of Waseda University and were approved (10K001). All mice were randomly divided into four groups: chow diet (control: Con), including chow diet, sedentary (*n* = 8) and chow diet plus exercise (Con + Ex, *n* = 9), ketogenic diet (KD), including KD, sedentary, *n* = 9, and KD plus exercise (KD + Ex, *n* = 9) groups. A KD diet TP-201450 (consisting of 76.1% fat, 8.9% protein and 3.5% carbohydrate, 7.342 kcal/g) and a chow diet AIN93G (consisting of 7% fat, 17.8% protein and 64.3% carbohydrate, 3.601 kcal/g) wt/wt were obtained from Trophic (TROPHIC Animal Feed High-tech Co., Ltd., Nantong, China). Mice were maintained on *ad libitum* chow diet or KD.

### 2.2. Endurance Capacity Test Protocol

One week before exhaustive exercise, all mice were accustomed to treadmill running at 15 m/min for 10 min. The endurance test was performed on a motorized treadmill (Natsume, Kyoto, Japan). That is, mice in the Con + Ex and KD + Ex groups were subjected to treadmill running at 10 m/min for 15 min, followed by 15 m/min and 20 m/min for 15 min each, and then 24 m/min and 7% grade until exhaustion. The protocol was approved by the Academic Research Ethical Review Committee. The exhaustion was defined as the inability to continue regular treadmill running despite the stimulation of repeated tapping on the back of the mouse. The running time of exercised mice was recorded. Immediately after the exhaustion, mice were terminated under light anesthesia with the inhalant isoflurane (Abbott, Tokyo, Japan). Blood samples were taken using heparin from the abdominal aorta under inhalant isoflurane-induced mild anesthesia, and tissues and organs were immediately excised and frozen in liquid nitrogen. Plasma was obtained from blood samples by centrifugation at 1500 *g* for 10 min at 4 °C. These samples were stored at −80 °C until analyses.

### 2.3. Plasma Biochemical Assessment

Plasma levels of glucose, non- esterified fatty acids (NEFA), triglyceride (TG), lipase, aspartate transaminase (AST), alanine transaminase (ALT), creatine kinase (CK), lactate dehydrogenase (LDH), blood urea nitrogen (BUN), cholesterol (CHO), high-density lipoprotein cholesterol (HDL), low-density lipoprotein cholesterol (LDL) and albumin were measured by Koutou-Biken Co. (Tsukuba, Japan). A commercial assay kit was employed to measure β-Hydroxybutyrate concentration (Cayman, MI, USA).

### 2.4. Statistical Analysis

Data are presented as means ± standard deviation (SD). For comparison of means between two groups, Student’s unpaired t-test was performed. A two-way analysis of variance (ANOVA) was performed to determine the main effects of diet and/or exercise. Statistical analysis was done using Graphpad 7.0 (Graphpad, Ltd., La Jolla, CA, USA). When this analysis revealed significant interaction, Tukey’s post-hoc test was performed to determine the significance among the means. Statistical significance was defined as *p* < 0.05.

## 3. Results and Discussion

### 3.1. Food Intake and Weight Change Following an 8-Week KD Diet

Consistent with previous findings, the weight of mice in KD groups became significantly lower after eight weeks compared with chow-feeding mice. The initial average weight of mice was 23.4 g, when they were eight weeks old after one-week adaptive feeding with the same chow, but after KD loading started, mice lose an average of 2.2 g compared to the initial weight in one week, and experience consisting weight loss when compared with mice in the chow groups. Since the first week, mice on chow feeding put on weight slowly, but KD feeding tended to maintain the same weight until their termination. At the end of the study, KD mice had an average weight of 21.8 g, whereas the mice on chow feeding was 27.1 g. KD mice dropped 7% of weight after eight-week KD feeding, whereas control put on 16% of their initial weight. When matched with the same older mice, KD mice consumed more calories, which did not correlate with weight gain. These results were consisting with other studies. In a 60-day KD feeding study, mice were 6.0 g lower in weight than the controls [9]. In a 12-week study, mice maintained on KD exhibited decreased weight compared with chow-fed diet, despite KD-feeding mice did intake much more calories [30]. Water consumption did not vary between groups (data not shown). In human trials, a meta-analysis analyzed thirteen trials and concluded that individuals assigned to a KD showed decreased body weight (weighted mean difference −0.91 (95% CI −1.65, −0.17) kg, 1415 patients) [31].

Dietary macronutrient composition remains a classic debate topic. KD is associated with weight loss and metabolic parameter improvement in obese objects, normal individuals and even athletes. Despite limited carbohydrate and protein consumption, the rodents tolerated this feeding well, and we did not observe abnormal phenomena during the study. Both feedings were provided *ad libitum*, and it was confirmed that KD-fed mice received adequate energy for daily life and were able to take an endurance test.

### 3.2. Absolute and Relative Tissue or Organ Weight of Animals

**Figure 1:**
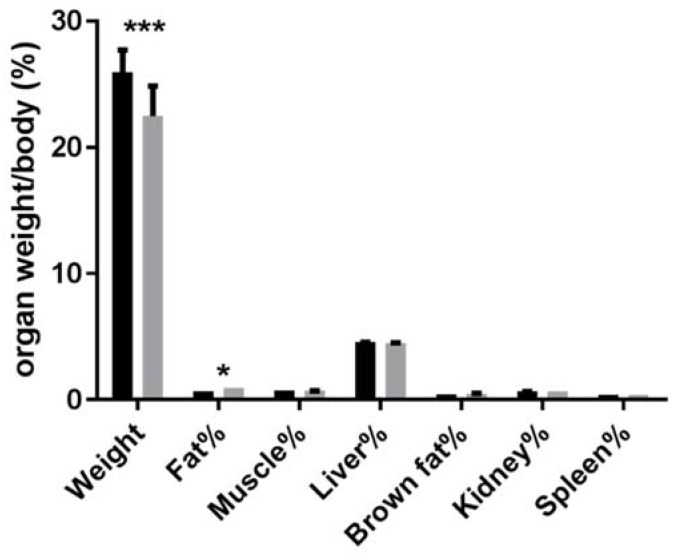
Organ mass/body mass rate of mice. * *p* < 0.05, *** *p* < 0.001 vs. chow. Fat: epididymal adipose tissue weight; muscle: gastrocnemius muscle tissue. Brown fat: brown adipose tissue. Fat, muscle and kidney were average of bilateral organ or tissue weight.

Absolute tissue or organ weight differed by diet effects. As shown in Figure 1, among them, fat (epididymal adipose tissue) of KD mice weighed more than the control group. Liver, muscle and kidney were lighter by KD than the control group. Brown fat (brown adipose tissue) and spleen did not differ significantly. The decrease in several organs or tissues may explain the decrease of total body weight. Garbow et al. reported no significant increase of body fat rate and a decrease in fat-free mass rate during a 12-week KD feeding under sedentary condition [31]. In another six-week KD study, rats subjected to a voluntary wheel running showed a significant decrease in epididymal adipose tissue weight, and this may be due to enhanced metabolism of KD [32]. The comparison of results from the present study and with previous studies leads us to suspect that KD combined with exercise training may obtain interesting results. The liver plays a key role in substrate availability and metabolism [33,34,35]. During fasting, the liver synthesizes glucose and metabolizes fatty acids, providing metabolic profiles. Hepatic metabolism is suspected to play an important part during KD-induced ketosis, which is usually considered to be similar to fasting. A high-fat diet like KD is always criticized for its risk to cause hepatic adipose infiltration. However, results here provide a low probability for lipidosis. Further investigation should be used to verify whether KD causes adipose infiltration.

### 3.3. Effect of KD on Endurance Exercise Performance in a Treadmill Running Test

**Figure 2:**
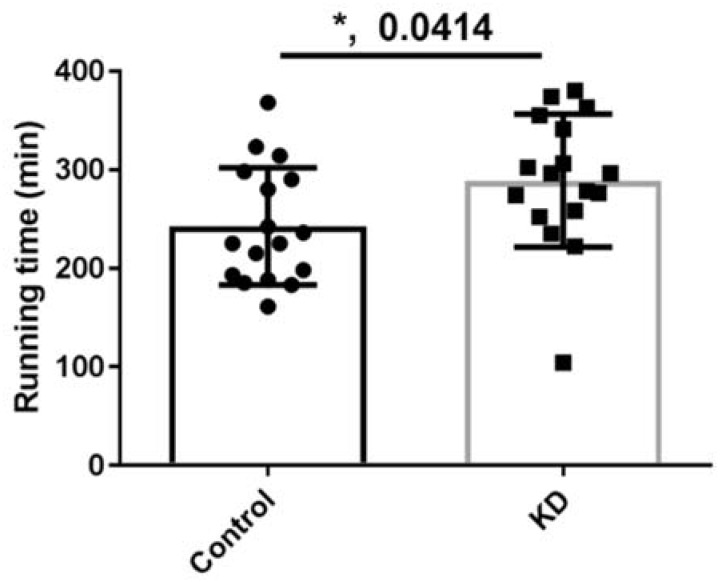
Performance of mice subjected to a chow-diet or ketogenic diet (KD). * *p* < 0.05, compared with the control group. *n* = 17 for each group.

As shown in Figure 2, the endurance capacity of mice subjected to the treadmill running test was significantly changed by KD. To decrease bias, the test was designed to be double-blind, meaning the experimenters did not determine the time taken to achieve exhaustion. As a result, mice subjected to control feeding ran up to 243 ± 60 min till exhausted, whereas KD-feeding mice achieved 289 ± 67 min. In our previous study, exhaustion in C57BL/6J fed by the chow-diet was approximately the same [36]. This consistency and stability enhanced the reproducibility of this study. In a recent review article of international society of sport nutrition stressed that KD-induced fat adaptation may increase the ability for body to use fat as fuel and should be given attention to for its performance-improving potential [37]. However, after reviewing an article about a protein and calorie-matched comparison of a KD and a Western diet model, this guide concluded that KD may be less effective for strength training. This may be due to the relatively low levels of protein in traditional KD to avoid gluconeogenesis. A modified KD diet that has limited amount of glucogenic amino acid contains more protein, thus solving this dilemma. It is suggested that increase of the lipid pool in adipose tissue might offer abundant fuel for endurance exercise. Another possibility would be increasing the utilization of lipids through the effects of lipoproteins. Based on these results and hypothesis, we screened targeted biochemistry analysis.

### 3.4. Effects of KD on Plasma Cholesterol, Glucose, NEFA, TG, LDL, HDL and β-Hydroxybutyrate Immediately after Endurance Exercise

**Figure 3:**
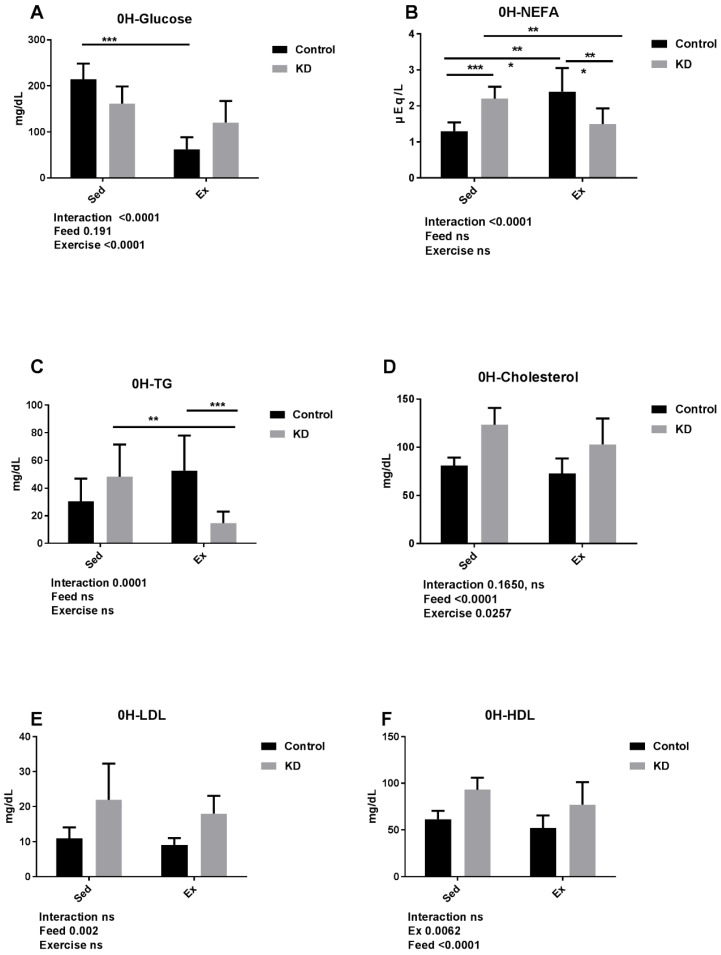
Plasma biochemistry results after KD feeding and immediately after exhaustion as indicated. A-F: Concentration of plasma glucose, non-esterified fatty acids (NEFA), triglyceride (TG), lipase, cholesterol (CHO), high-density lipoprotein cholesterol (HDL) and low-density lipoprotein cholesterol (LDL). * *p* < 0.05, ** *p* < 0.01 and *** *p* < 0.001.

As shown in Figure 3, glucose was significantly decreased by exercise in the chow-feeding group, but not in the KD group. This might be attributed to the low coefficient of utilization in the KD mice, which mainly maintained a supply system largely powered by fat.

Non-esterified fatty acids (NEFA) were elevated by exercise in the control group but decreased by exercise in the KD group. Additionally, during base line, NEFA concentration is elevated by KD, and was significantly lower after exercise in the KD group compared with chow. Immediately after exercise, the concentration of TG was significantly lower in the KD group. These results combined showed that FFA and TG were utilized sufficiently during exercise in the KD group [38,39,40].

CHO, LDL and HDL were significantly elevated by KD, and CHO and LDL were increased by exercise. LDL and HDL are components of CHO. There is a direct relationship between chronically elevated LDL levels (dyslipidemia) and coronary heart disease. LDL is prone to be oxidized, and excessively oxidized LDL may cause aggregation and precipitation in the artery, leading to arteriosclerosis. HDL removes cholesterol to the liver, and radiography studies have shown that a high content of HDL may predict a low probability of vascular lumen stenosis. Recent studies have carefully examined how exercise alone could lower CHO and LDL. First, exercise stimulates enzymes that help move LDL from the blood (and blood-vessel walls) to the liver. From there, the cholesterol is converted into bile (for digestion) or excreted. Second, exercise increases the size of the lipoproteins and finally LDL is turned into HDL. Several studies reported that KD resulted in high CHO, although our results showed that KD were involved with the elevation of LDL and HDL, both components of CHO [41,42].

β-Hydroxybutyrate mainly comes from the oxidation of fatty acids and is exported to peripheral tissues for energy supply. Accounting for approximately 75% of the ketone bodies in blood, the significant higher concentration of β-Hydroxybutyrate in Ex and KD groups indicated utilization of fat or ketosis [8,33]. As shown in Table 1, elevated concentration of β-Hydroxybutyrate indicates that mice were exercised to exhaustion, whereas after exhaustive exercise, the concentration of β-Hydroxybutyrate in KD feeding group decreased significantly, indicating the capacity of keto-adapted mouse to utilize ketone bodies.

### 3.5. Effects of KD on Plasma Albumin, AST, ALT, Lipase, Amylase, CK, LDH, UA and BUN Immediately after Endurance Exercise

**Figure 4:**
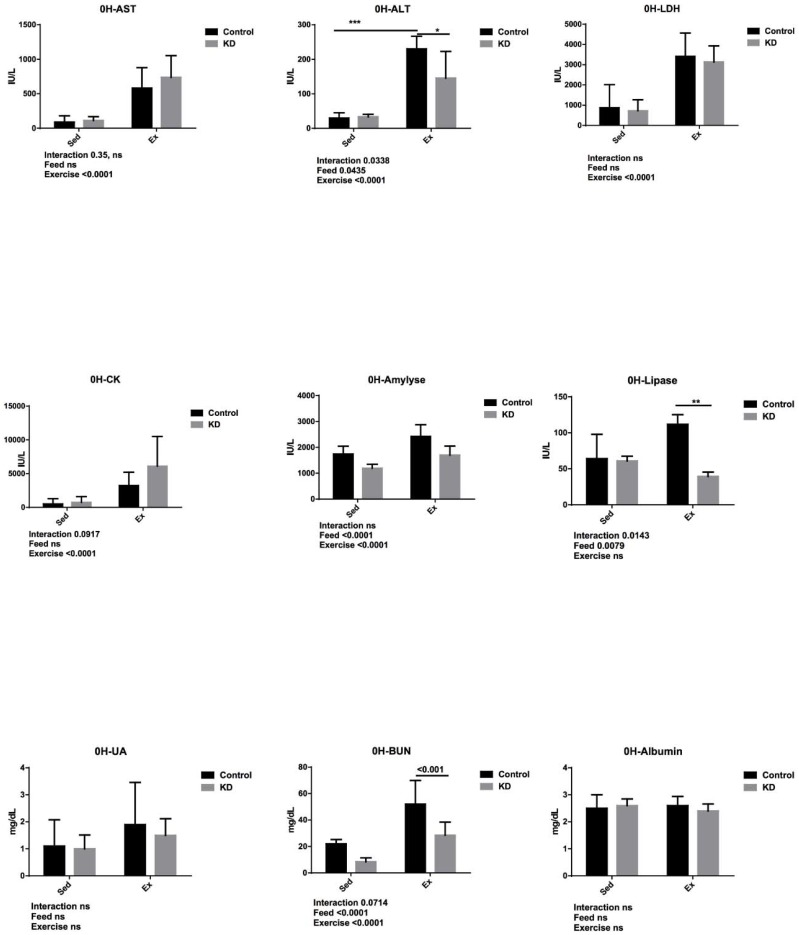
Plasma biochemistry results after KD feeding and immediately after exhaustion as indicated. * *p* < 0.05, ** *p* < 0.01, *** *p* < 0.001.

Effects of KD on plasma albumin, AST, ALT, lipase, amylase, CK, LDH, UA and BUN immediately after endurance exercise were shown in Figure 4. AST, also known as glutamic oxaloacetic transaminase (GOT), and ALT, also known as glutamic pyruvic transaminase (GPT) were employed as hepatic damage markers [43,44,45,46]. They were both elevated by exercise, and in the post-exercise stage, ALT was significantly decreased by KD, indicating that KD may contribute to protecting the liver from exercise-induced hepatic damage. Several studies reported that long-term KD may cause endoplasmic reticulum (ER) stress and apoptosis. In our study, however, KD did not undermine hepatic health, apparently being dependent on the results obtained by other studies [31,33,34].

Lipase and amylase are enzymes which catalyze fat or starch into NEFA, glycerol and glucose. Lipase was decreased by KD. During high-fat feeding, demands for an effective fat utilization system are important. This may strengthen the transportation of fat and NEFA or ketone bodies by blood and into organs and tissues that are important in both catabolism and synthesis such as liver and muscle. Due to keto-adaptation, some organs or tissues are forced to utilize ketone bodies, since glucose fails to sustain the energy supply. NEFAs are transported by binding to albumin in the blood, and they are re-synthesized into TG as a pool, safe to store, and readily available. In the muscle tissue, it is called intramuscular TG (IMTG), where TG is accumulated into little lipid drop, and lipases including adipose triglyceride lipase (ATGL) and hormone sensitive lipase (HSL) conjunction with the lipid drops catalyze them into NEFAs and glycerol [47,48,49,50]. Keto-adapted mice possess an enhanced ability in fat-utilization, including metabolism, catabolism and transportation, allowing them to increase their exercise endurance. It may be interesting to study the underlying molecular mechanisms among keto-adaptation during exercise.

Amylase was enhanced by exercise but decreased by KD. As discussed above, a restricted carbohydrate supply and abundant fat supply combine to cause keto-adaptation. This adaptation mechanism weakened the glucose-powered energy supply system. The weakened role of amylase may result in its low concentration in the blood. Exercise demands energy, which may be why amylase increases in the blood during exercise, giving the credit for strengthened secretion function of this enzyme in the pancreas. Permeability of various organs rises during endurance exercise, and that may be another possible cause for elevated amylase concentration [51,52].

Creatine kinase (CK), also known as creatine phosphor kinase (CPK) and lactate dehydrogenase (LDH) were employed as muscle damage markers. In the present study, they both increased due to exercise, and thus KD failed to protect muscle damage. However, decreased absolute weight did not worsen the damage, according to our results. This may be a useful result for endurance athletes such as those in marathons or long-distance cycling, since muscle damage is common among such athletes. However, the practicability of these studies in athletes needs to be investigated further.

BUN was employed as a kidney injury marker, as well as an indicator of exercise tolerance. At the same time, it is also a protein degradation marker [53,54]. BUN was significantly lower in KD groups, but this effect was enhanced by exercise. This may be partly attributed to the lower protein content in the KD. Studies on exercise-induced organ damage showed that during exhaustive exercise, acute kidney injury or acute renal failure may occur. Whether the KD has the potential to protect the kidney from renal damage may be an interesting point for further investigation. A study conducted this year claimed that a 21 d-KD did not affect the acid-base status in elite athletes, indicating that KD had a minimum effect on renal function even during constant use [55]. Although these indicators were not measured in this study, we should clarify that KIM-1, creatine and acid-base status are also markers for exercise-induced renal injury, and future studies should focus on them to find out whether KD could potentially change these markers.

Results in this part clarified how KD interacted with blood biomarker alterations during endurance exercise. The network of cytokines, chemokines, myokines and adipokines interact closely and in a complex way with exercise and inflammation [56,57]. Further investigations need to be undertaken.

### 3.6. Correlations among Running Time and Weight, Blood NEFA, Amylase or Lipase

**Figure 5:**
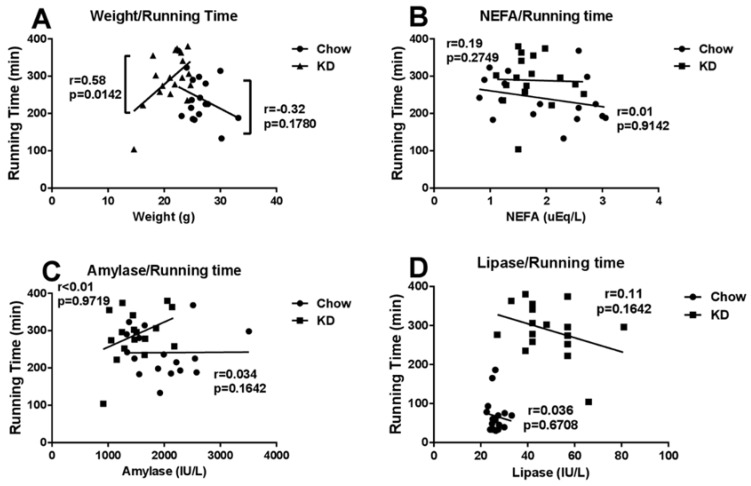
Correlations between (**A**) mice weight and running time, (**B**) blood non- esterified fatty acids (NEFA) concentration and running time, (**C**) blood amylase concentration and running time, and (**D**) blood lipase concentration and running time. *p* < 0.05 is found between KD mice weight and KD running time.

As shown in Figure 5, the relation between weight and exercise performance shows different patterns between chow and KD groups. The smaller the mice are, the more ‘time to exhaustion’ increased, but here we found no significance. KD-feeding mice were generally smaller than chow-feeding mice, and surprisingly, we found more significance here in relation to their weight. The exercise capacity is prolonged when subject is relatively bigger. We considered the weight of the KD mice alongside their capacity to utilize fat and ketone body, and this increased compared to chow-feeding subjects. However, KD may not apply to every individual, since low-weight subjects showed low fat adaptation ability, and had worse endurance. Blood NEFA and amylase failed to be associated with exercise capacity. However, lipase concentration declined when matched with running time in both groups, though the starting concentration greatly differed between groups. This could be due to the increasing demand of fat during prolonged exercise, therefore blood lipase was transported and absorbed into the interspace between muscle fibers and adipocytes, hydrolyzing TG stored in the muscular pool (IMTG) or adipocyte fat pool, thus resulting in a decrease in the blood. Since there are abundant studies reporting ALT, AST, LDH and CK being closely correlated with exercise time, thus, this will not be elaborated about it here. In another animal study, short- and long-term KD-feeding improved several liver oxidative stress markers, showing that the anti-oxidative potential of KD may also enhance endurance exercise [58]. Muscular fat oxidation capacity may also play a key role during endurance exercise, and the mechanism of this needs further study [59].

## 4. Conclusions

In this study, an eight-week ketogenic high-fat, low-carbohydrate diet increased the capacity of endurance exercise in mice without aggravating muscle injury, despite the decrease of absolute muscle volume. The potential mechanism is most possibly the enhanced ability to transport and metabolize fat. Apart from this, KD showed potential to protect liver and kidney from acute exercise-induced injuries. These data suggest that KD may contribute to prolonged-exercise capacity.

## Figures and Tables

**Table 1:** Effect of KD feeding and/or exercise on β-Hydroxybutyrate concentration.

	Con	Ex	KD	KD + Ex
β-Hydroxybutyrate, mmol/L	0.29 ± 0.038 b,c	2.8 ± 0.52 a,d	2.4 ± 0.64 a,d	0.72 ± 0.10 b,c

Data are presented as means ± SDs. *p* < 0.05 compared with Con(a)-, Ex(b)-, KD(c)- and KD + Ex(d). Con, Ex, KD and KD + Ex stands for chow die, chow diet plus exercise, ketogenic diet and ketogenic diet plus exercise. a, significantly different from Con; b, significantly different from Ex; c, significantly different from KD; d, significantly different from KD + Ex.
